# Adoption and Implementation of California State Transgender and Nonbinary Protections in Los Angeles Area High Schools

**DOI:** 10.1111/josh.70100

**Published:** 2025-12-10

**Authors:** Rory P. O'Brien, Kevin Yu, Julie A. Cederbaum, Laura Ferguson, Jeremy T. Goldbach, Harmony Rhoades, John R. Blosnich

**Affiliations:** ^1^ Suzanne Dworak‐Peck School of Social Work University of Southern California Los Angeles California USA; ^2^ School of Social Work San Diego State University San Diego California USA; ^3^ Michigan State University School of Social Work East Lansing Michigan USA; ^4^ Institute on Inequalities in Global Health University of Southern California Los Angeles California USA; ^5^ George Warren Brown School of Social Work Washington University in St. Louis St Louis Missouri USA

**Keywords:** adolescents, case study, school policy, transgender and nonbinary

## Abstract

**Background:**

California is a leader in adopting numerous transgender and nonbinary adolescent (TNBA)‐protective education policies. However, the adoption and implementation of these policies remain largely unexamined. This study explored local school adoption and implementation of California state TNBA‐protective policies.

**Methods:**

Nine Los Angeles area high schools enrolled in another study (R01MD016082) participated in this explorative qualitative case study. Case study methods included document collection, campus observations, member checks, and seven student focus group discussions (FGDs; *n* = 39) between August 2022 and April 2024. Data were analyzed by multiple coders following the Framework Method to compare the implementation of school, district, and state policies.

**Results:**

Implementation of name changes, gender‐neutral restrooms, private accommodations, and sexual health education varied between schools and districts. While many schools did not meet state mandates to protect TNBA student access to education, others feasibly implemented these policies, and some adopted innovative approaches to protect TNBA. District oversight, publication of policies and facility access, staff training, funding, and infrastructure were key factors in successful implementation.

**Implications for School Health Policy, Practice, and Equity:**

Study findings highlight the feasibility of the implementation of TNBA protections in schools, with recommendations to adopt gender transition planning, publish policies for student access, and ensure the availability of gender‐neutral restrooms and private accommodations.

**Conclusions:**

Implementation of these policies is feasible with sufficient support and oversight. TNBA‐protective policies can be strengthened with accountability mechanisms and promotion of implementation strategies, such as technical assistance. Future research should examine the implementation of these policies statewide.

**Abbreviations**
FGD: focus group discussions
TNBA: transgender and nonbinary adolescents

## 1 Introduction

Transgender and nonbinary adolescents (TNBA), compared to cisgender peers [1, 2, 3], face educational barriers in the United States, including difficulties updating records, having gender identities respected, and restricted restroom access [1, 4, 5]. TNBA also face increased risk of violence compared to cisgender peers [2, 6], and unique forms of hostility such as misgendering and forcible disclosure of their gender identities [7, 8]. Exposures to and anticipation of violence are linked to increased truancy [9], harsh discipline [10], school dropout [11], substance use [2], and suicide attempt [2, 12] for TNBA. School policies that protect TNBA privacy, self‐determination, and equitable access are essential to promoting their academic success and health.

U.S. state and local governments increasingly diverge on youth rights, with some protecting TNBA rights [13, 14, 15, 16] while others adopt hostile policies to forcibly out students [17], restrict access to facilities and sports [18, 19], ban books about gender diversity [20], and prohibit inclusive instruction [21]. California leads in passing protective legislation, including records, activity, and facility nondiscrimination in the School Success and Opportunity Act (SSOA) [22] and comprehensive sexual health education requirements in the California Healthy Youth Act (CHYA) [23].

Specifically, California affirms TNBA freedom from discrimination, bullying, and harassment based on gender identity and rights to use sex‐segregated facilities and participate in sex‐segregated activities consistent with students' gender identities [22, 24]. The California School Board Association's legal guidance interprets the SSOA to require that schools update data systems to permit student unofficial name changes and provide private changing facilities for students upon request [25]. Unofficial name changes include student‐ or parent‐asserted non‐legal requests to amend non‐legal documents, such as directory information, as opposed to official court‐mandated changes that can result in amended legal documents, like birth certificates. The Equal Restroom Access Act (ERAA) additionally mandates that public single‐stall restrooms in California be all‐gender [26]. The CHYA mandates that public schools provide 7–12th grade students with medically accurate and age‐appropriate instruction on sexual health, including on gender identity and sexual orientation [23]. The SSOA and ERAA required that students be permitted to use restrooms consistent with gender identity and that single‐stall restrooms be all‐gender; a new state law further requires that schools provide all‐gender restrooms by 2026 [27]. While protective, these laws have limitations. The CHYA does not set a minimum number of hours of instruction or requirements for how to deliver curriculum (e.g., in a class or single event), so schools likely vary in hours of instruction.

The promotion of an inclusive school climate is a protective factor for TNBA mental and academic outcomes [28], yet, nationwide, less than half of students report that their school has TNBA‐protective policies [29, 30]. Although California has adopted TNBA‐protective laws, few studies have assessed their adoption and implementation. Among those that have, one found that the positive effects of inclusive curriculum—that is, curriculum that affirmatively addresses sexual and gender diversity—on California high school climate are evident when it is delivered by many teachers, as compared to schools where fewer teachers deliver inclusive curriculum [31]. Another study in San Francisco identified variability in the adoption of state anti‐bullying and nondiscrimination policies, but did not investigate implementation [16].

Teachers, staff, and school leaders play vital roles in shaping students' experiences; TNBA who report having at least one supportive adult at school also report feeling safer and a greater sense of belonging [30]. Teachers' and principals' attitudes toward TNBA have been found to be favorable in regional studies [32, 33] and neutral to slightly favorable in nationally representative studies [30, 34, 35]. However, favorable attitudes alone do not ensure successful policy implementation, which involves specific knowledge, skills, resources, and changes in infrastructure. Additionally, these favorable attitudes may result in yet‐unknown innovations beyond the requirements of the law.

School staff, including teachers and administrators, engage in discrete decision‐making that shapes how laws function, such that regulations and their implementation vary locally [36]. “Street‐level bureaucrats”—those workers in highly rule‐bound service settings who implement policy on‐the‐ground [37]—play a key role in policy implementation in public schools. Their interpretation of policy is shaped by factors such as personal beliefs, knowledge, institutional norms, practical considerations, and perceptions of students [36]. Implementation of these policies may vary according to realities in each particular school, such as staff beliefs and knowledge, funding, and community norms [36, 38, 39]. Local decision‐making, therefore, allows for substantial variation across districts and schools, highlighting the need for continued research.

Overall, little is known about local adoption and implementation of TNBA‐protective policies. Evidence of their implementation is needed to strengthen these policies and support their positive impacts locally and diffusion nationwide. Specifically, examination of local policy implementation can identify pitfalls and innovations to address in policy efforts in California and elsewhere. This multiple case study investigates and compares the adoption and implementation of TNBA‐protective California policies (e.g., written school rules), facilities (e.g., restrooms), and procedures (e.g., enactment of school rules) in nine public high schools in Los Angeles County.

## 2 Methods

### 2.1 Study Design and Participants

This exploratory qualitative case study enrolled nine schools within a larger NIH‐funded randomized controlled trial (RCT; R01MD016082). Case study eligibility included RCT enrollment, location in Los Angeles County, and agreement to an IRB‐approved waiver of parental consent. RCT staff recruited schools to participate and then introduced school staff (hereafter, liaisons) to this study. Liaisons facilitated access to data, but were not research‐engaged or participants and were not asked to consent to any study procedures. The University of Southern California Internal Review Board approved all study protocols. Data collection co‐occurred with the first year of the RCT, including a Fall semester 10‐week psychosocial intervention with sexual and gender minority students and a Spring semester 5‐week social justice youth development intervention with 10–20 students. Given the small number of RCT participants and the Fall interventions' focus on individual‐level change, RCT activities are not expected to have had any appreciable effect on school policies, procedures, and facilities during this study's data collection.

Nine schools across five districts participated in this study over 2 years. Table 1 details schools and districts (pseudonymized for confidentiality), liaison roles, and student body demographics.

**TABLE 1:** Schools, school characteristics, and liaison roles.

Year	District	School	Liaison roles (*n*, if more than 1)	Student body size	% Students of color	% Students eligible for reduced‐price lunch
2022–2023	Aster	Dandelion	Counselors (2)	+1700	80%–90%	50%–60%
		Dahlia	Librarian/former GSA Advisor	+1000	80%–90%	50%–60%
		Thistle	Counselors (2)	+500	80%–90%	50%–60%
	Amaryllis	Agapanthus	Counselors/GSA Advisors (2), Interventionist	+1600	90%–100%	70%–80%
		Daffodil	Teachers/GSA Advisors (2), Activities Director	+2100	90%–100%	70%–80%
2023–2024	Buttercup	Delphinium	Teachers/GSA Advisors (2)	+3600	80%–90%	50%–60%
		Marigold	Counselor, Teachers (2)	+3500	60%–70%	30%–40%
	Mallow	Hibiscus	Counselor, Teacher	+1700	90%–100%	90%–100%
	Lily	Tiger	Teachers/GSA Advisors (2), Assistant Principal	+2600	60%–70%	20%–30%

*Note:* Student body size, percentage of students of color (African American, American Indian or Alaska Native, Asian, Filipino, Hispanic or Latino, Pacific Islander, and/or Two or more races per California Department of Education School Profile categories), and percentage of students eligible for reduced‐price lunch are based on available statistics reported on the California Department of Education School Profile Data at the time of data collection. Approximations of these three school‐level characteristics are presented to prevent identification of schools.

Abbreviation: GSA, gender and sexuality alliance.

### 2.2 Data Collection Methods

The multiple case study approach prioritizes collecting many data types to develop rich case characterizations of each school and then compare across cases [40]. Documents, observations, and member checks were primary study data sources. These methods assessed adoption and implementation of California legal requirements, such as health education delivery [23], posting of gender‐inclusive nondiscrimination policies [24], and name changes [25]. For example, researchers assessed name change procedures by examining student handbooks and registrar policies (documents) and by speaking and emailing with registrars, counselors, and principals (observations, member checks). Focus group discussions (FGDs), described below, were incorporated as an additional primary data source in three schools.

#### 2.2.1 Documents

The first author introduced liaisons to the study via web‐conferencing and requested their help accessing school documents, including handbooks, course listings, syllabi, registrar policies, school reports, and other TNBA‐related policies. We specifically requested documents that were likely to include information on school adoption and implementation of policies. For example, course listings, health syllabi, curriculum, and School Accountability Report Cards (which list schools' instructional materials per subject) were reviewed to determine if and how schools delivered sexual health curriculum. Liaisons provided documents via email, e‐introduced researchers to colleagues, and provided paper copies during observations. Researchers also downloaded documents directly from school websites.

#### 2.2.2 Observations

Liaisons helped plan and guide researchers during campus observations, which lasted between 45 and 150 min. When possible, two researchers (first author and colleague) conducted observations. Observations occurred before and after school when students were less likely to be present. Researchers arrived at the main entrance to meet with liaisons, who guided researchers to all locations included in the observation protocol. The protocol included sections on gender‐neutral restrooms (GNRs) and locker rooms, gender and sexuality alliances, campus offices, libraries, and the broader campus. Note sections, such as for GNRs, required specification of the number and locations of GNRs, signage, and whether access required a key or staff permission, among other questions. Observers took notes and photographs to document the implementation of California legal requirements, such as public posting of nondiscrimination policies, and sought to gather as much rich detail of campus climate and facilities as possible in line with the case study approach. Photographs did not include people. Observers retyped handwritten notes and captioned photographs immediately after observations.

#### 2.2.3 Member Checks

Data collection and analysis sometimes revealed gaps and a need for clarification of findings. “Member checking” is a method of seeking further information, reflection, and feedback on the data; the purpose of member checking in this study was not to seek validation, but rather to explore persisting uncertainties and create opportunity for co‐construction of preliminary write‐ups of school findings [41]. Two types of member checks were conducted with school liaisons. Given the explorative nature of the study, the first author followed up with liaisons with clarifying questions and to pursue leads throughout data collection and preliminary analysis (August 2022–May 2024). The first author also composed brief case characterizations for each school and requested liaisons' feedback and reflections on those characterizations (April–August 2023).

#### 2.2.4 Focus Group Discussions

Researchers invited student advocates at Dandelion, Dahlia, and Agapanthus High Schools (intervention schools in the RCT study) to join FGDs in late Spring 2023. School liaisons referred students who had participated in the RCT intervention to participate in the FGDs, which included sexual and gender minority students and heterosexual cisgender student allies. FGD participants provided informed assent before FGDs; the IRB‐approved a waiver of parental consent for this study. Students each received $25 cash compensation after participating.

FGDs lasted 45–60 min in classrooms during school, with administrator clearance for attendance. FGDs included 2–8 participants, totaling 39 participants across 7 groups where participants discussed their advocacy experiences on campus. Of the 39 participants, 11 identified as TNBA. A secure third‐party company transcribed FGD audio files.

FGDs sought to answer a separate research question on student advocacy experiences. Participants were not asked to report on policies or facilities, but they nonetheless shared information that contextualized findings from documents, observations, and member checks.

### 2.3 Data Analysis

The first author co‐analyzed data in Atlas.ti with support from multiple coders. Four co‐coders collaboratively coded and analyzed the data and were all LGBTQA+ (nonbinary queer, cisgender queer woman, cisgender gay man), of varied racial backgrounds (White, biracial Black/White, Asian), and with different academic experience, including two MSW graduates, a PhD student, and a postdoctoral fellow. We utilized the Framework Method, originating from social policy analysis, which involves data familiarization, coding, analytic framework development, and charting and interpreting results [42]. The Framework Method facilitates case comparisons by producing spreadsheets with rows (cases) and columns (policies). Documents, observation notes, and photographs, and member check responses (all data except the FGD transcripts) were compiled into a common dataset for framework analysis. An a priori codebook reflected California policies with superordinate codes, such as Title IX, Privacy, and CHYA, with subcodes reflecting specific requirements, such as the Nondiscrimination subcodes, “Posted on campus,” and “Specifies complaint procedure.” Co‐coders independently coded data and met semi‐weekly. Coders' data were charted in Excel, where differences were discussed and resolved before conducting line‐by‐line comparisons across schools, districts, and state policies. FGD data were separately analyzed for another research question using a thematic analytical approach [43]. FGD data were incorporated into the framework chart as relevant (e.g., when students incidentally shared insights related to school resources and infrastructure, such as the accessibility of gender‐neutral restrooms) to facilitate triangulation between multiple data sources. The clear analytical approach, flexible but clearly defined codebook, and use of co‐coders promoted analytical rigor. The scope of data collection, multivocality, and member checking and liaison feedback on case characterizations supported the trustworthiness of the data.

## 3 Results

Study findings are presented in five sections: student records, GNRs, private accommodations, sexual health education, and innovations. Data on nondiscrimination, anti‐bullying, and Title IX are not reviewed in detail because, while schools varied in posting policies on campus, all schools specified gender identity as a protected class in handbooks and websites. The sections below focus on policies that varied most across schools. Table 2 overviews findings per school and Table S1 reviews the common data types collected and observed in each school.

**TABLE 2:** Findings by school.

District	School	Name changes	Gender‐neutral restrooms (*n*; description)	Accommodations (not including toilet stalls and GNRs)	Health education
State Laws and California School Board Association Legal Guidance		(a) Data systems can be updated to reflect names, (b) Schools protect student records privacy, (c) Parent permission not required for unofficial name changes	(a) Single‐stall restrooms are all gender‐neutral, (b) students can access restrooms consistent with gender identity	Students may request private accommodations upon request	(a) School delivers comprehensive sexual health education, (b) law does not specify amount of instruction
Aster	Dandelion	Some staff agree to add aliases, some do not. Aliases do not transfer to attendance rosters or email	1: single stall in nurse's office; permission required	Provided, external to gendered locker rooms	Unconfirmed potential delivery of district‐approved in 10th grade history
	Thistle	Adds Aliases upon request. Aliases do not transfer to attendance rosters or email	3: single stall each in nurse's office, admin office, and middle school nurse's office; permission required	None in boys' locker; privacy stalls in girls' locker	10 weeks of district‐approved curriculum delivered, split between world history and chemistry courses
	Dahlia	Adds Aliases upon request. Aliases do not transfer to attendance rosters or email	2: single stall each in nurse's office and main hall; permission required	None in boys' locker; privacy stalls in girls' locker	District‐approved curriculum not delivered; school provides 10th grade “boosters” events
Amaryllis	Daffodil	No name change process, students must ask counselors to notify teachers semesterly	3: single stall each in nurse's office and in main area (×2); permission required	Not provided	Curriculum delivered within 9th grade biology course, duration unspecified
	Agapanthus	No name change process, students must ask counselors to notify teachers semesterly	1: single stall in nurse's office; permission required	Not provided	3–4 week curriculum delivered in 9th grade biology course
Buttercup	Delphinium	Gender transition planning, parent permission not required, email updates not specified	1: multi‐stall in main area; no permission required	Gym facilities under construction. Plans to build entirely gender‐neutral locker facilities	Unconfirmed delivery of district‐approved curriculum; district mandates reporting on implementation
	Marigold	Gender transition planning, parent permission not required, email updates not specified	1: single stall in nurse's office; permission required	Not provided	4 week district‐approved curriculum delivered in 9th grade biology; district mandates reporting on implementation
Mallow	Hibiscus	Gender transition planning, parent permission not required, email updates facilitated	2: single stall each in nurse's office and main area; permission required	Provided, external to gendered locker rooms	Health course provided and required for graduation
Lily	Tiger	Gender transition planning, parent permission required, email updates not specified	3: single stall each in nurse's office, main hall, and library; permission required	Provided, external to gendered locker rooms	Health course provided and required for graduation; Health rallies listed on school calendar

At the district level, the Buttercup, Lily, and Mallow Districts had adopted and implemented more TNBA protections than the Aster and Amaryllis Districts. The Buttercup, Lily, and Mallow Districts had robust name change procedures and sexual health curriculum. The Lily and Mallow Districts provided centrally located GNRs outside of the nurse's office and private accommodations near their gyms. The Aster District had a name change policy and district‐approved sexual health curriculum, but its implementation by Aster schools was highly inconsistent. The Amaryllis District had no apparent district‐wide name change policy, did not provide private accommodations, and did not have a district‐adopted sexual health curriculum.

### 3.1 Student Records

Four districts—all but Amaryllis—had transgender student policies that reflected California's SSOA [22, 44]. Three of those districts, Buttercup, Lily, and Mallow, used gender transition plans, described below, whereas the Aster District had a transgender student policy but did not offer transition planning. Schools also varied in their email policies for names that have been changed. Schools used multiple systems for records, classroom management, and email; name changes did not necessarily transfer between them. Email addresses composed of or searchable by legal names (*Legal Name* <*email>*) can out TNBA, yet most schools reported no procedures to update emails.

#### 3.1.1 Gender Transition Planning

**FIGURE 1:**
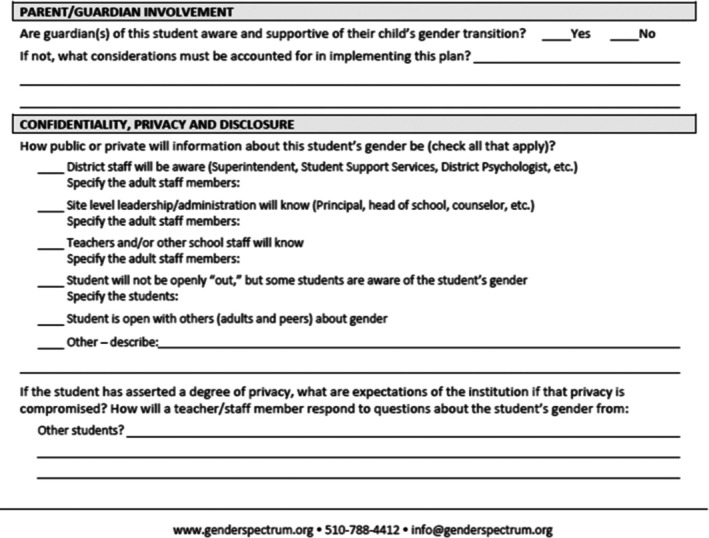
A portion of the Buttercup District gender transition plan.

Delphinium and Marigold Highs (Buttercup District), Hibiscus (Mallow District), and Tiger (Lily District) provided copies of their “Gender Transition Plans” (see Figure 1). Originally disseminated by the organization, Gender Spectrum, Gender Transition Plans facilitate student privacy and self‐determination in names, gender markers, and facility access. They generally include sections on privacy (who can know about the student's gender identity), safety, where name changes are needed (e.g., attendance, gradebooks, email), facilities, activities, and action steps. Delphinium, Marigold, and Hibiscus allowed name changes without parent permission, whereas Tiger required at least one parent's permission. Parents at all schools could view name changes via online portals; liaisons stated during observations that staff would counsel students on managing visibility during transition planning. Transition plans indicated that students could alternatively request unwritten staff notifications (e.g., that individual teachers be verbally informed of the student's name and gender), instead of making written parent‐viewable changes. Of schools with gender transition planning, only Hibiscus's plan specifically asked students if they wanted email addresses changed.

#### 3.1.2 Aliases

**FIGURE 2:**
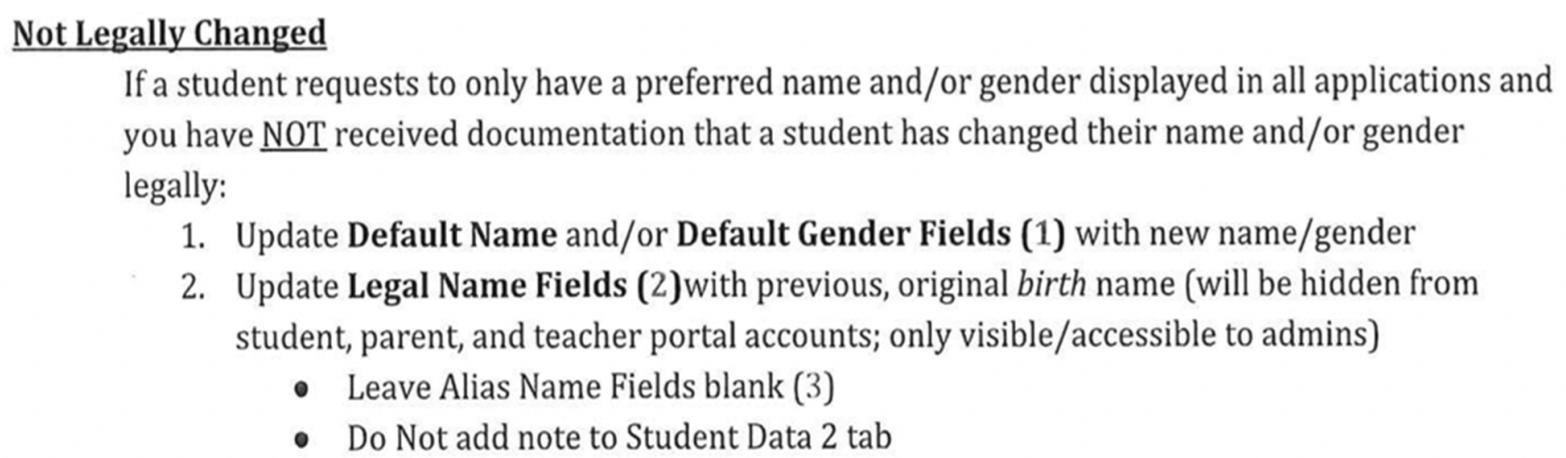
Unused Aster District guidance on non‐legal name changes.

Aster District instructions, provided via email by the Dahlia registrar, detailed how to make unofficial name changes to protect student privacy, even from parents' view (see Figure 2). Aster District training documents instructed schools to respond to requests by moving the student's legal name from the “Default” field to the “Legal” field, entering the student's asserted name into the now‐empty “Default” field, and leaving the “Alias” (e.g., nickname) field empty. However, no Aster District school followed these instructions. Instead, they retained legal names in the “Default” field and added asserted names to the “Alias” field, such that students' names appeared in online portals as *Legal‐First* “Alias” *Legal‐Last‐Name*. In addition to retaining visible legal names, researchers observed that aliases did not transfer to printed attendance rosters; both of these factors risk violations of privacy and misgendering.

Aster District students reported challenges accessing name changes. Thistle FGD participants shared that requesting name changes is “a hassle,” where even supportive parents need to devote substantial time. Another student added, “They gotta call and call and call.” Such advocacy can be particularly difficult for parents lacking resources, including single parents and parents facing racial and economic oppression. Whereas Dandelion counselors also added aliases, the Dandelion registrar erroneously claimed that name changes were illegal. Highlighting these inconsistencies, a Dandelion FGD participant stated that requesting a name change felt “Like a speak‐easy, you, like, have to know … the right people to talk to and … the right places to go. It's a mess.” The unused Aster District registrar instructions also detailed email change instructions. While Dandelion and Thistle FGD participants said that they could add pronouns in the classroom management software, a Thistle student noted that “Whenever you have to share a file…or email someone, you have to use their deadname.” Not updating email addresses per district policy resulted in students having to misgender each other as part of their daily education.

#### 3.1.3 Semesterly Requests

The Amaryllis District and its schools lacked any name change policy. Except for legal name changes, Daffodil and Agapanthus students had to ask counselors to notify teachers of their names each semester, akin to the unwritten communications provided as an alternative in districts with gender transition planning. Alone, this procedure did not meet state requirements that schools update data systems to allow name changes and risked misgendering by other staff, including substitutes and administrators.

### 3.2 Gender‐Neutral Restrooms

California schools must allow students to access facilities according to their gender identity [44]. All schools provided one to three GNRs, most of which were single‐stall facilities located in monitored areas, like nurse's offices, and required staff permission or keys to access. There were no clear district‐level patterns in the number of GNRs offered; for example, the number of observed GNRs in a school was not necessarily proportionate to the number of enrolled students (see Table 1 for student body size per school). In all three schools where FGDs were held, participants learned for the first time of GNRs in nurse's offices, with a Thistle student noting that, even if they tried to use the GNR, “The nurse is always on their lunch break, like, always.” GNR location and reliance on specific staff reportedly reduced access.

Dahlia, Daffodil, Hibiscus, and Tiger each had a nurse's office GNR and centrally located GNR(s). However, Dahlia, Agapanthus, and Hibiscus liaisons noted that locating GNRs in less supervised spaces posed challenges to controlling drug, sex, and bullying activities. During observation, Hibiscus liaisons and an administrator shared that their centrally located GNR was initially unlocked. After students smoked in it, liaisons advocated, despite the administrators' opposition, to require student IDs and an administrator key for access.

Accessing Dahlia's two GNRs, off a hallway and in the nurse's office, also proved challenging. Staff struggled to locate the hallway GNR during the observation; ultimately, an administrator guided them. Its sign still designated “Staff,” which was covered by another sign that read “Out of order until further notice.” This reflected Dahlia's School Plan for Student Achievement, which acknowledged that “Our restrooms are in desperate need of upgrade,” citing frequent plumbing issues. The Dahlia nurse's GNR was located downstairs; when asked about accessibility, the liaison stated that the elevator does not always work. The Dahlia nurse also cited cleanliness concerns when stating that she does not permit boys to use the GNR. When asked, she replied that LGBTQ students could use the GNR, but this nonetheless revealed that the nurse's assumptions of students' gender and sexuality determined GNR access. Overall, observation revealed that access to Dahlia GNRs was constrained by funding and maintenance issues, ability, and staff perception of student gender.

Daffodil provided two single‐stall GNRs located alongside single‐stall gendered boys' and girls' restrooms. This differed from other schools, where GNRs were often in separate and supervised spaces apart from gendered facilities. While a more equal setup, the relative number of GNRs and gendered restrooms campuswide nonetheless posed challenges. The GNRs were a 6‐min walk from the furthest classroom; the 6‐min passing period meant that TNBA must choose between biological functions and tardiness.

Delphinium was unique in providing one centrally located, unlocked, multiple‐stall GNR. This facility was unlocked, did not require staff permission for use, and featured locking stall doors and floor‐to‐ceiling stall privacy walls. However, as the sole GNR on a large campus, the furthest classrooms were 10 min' walk away, risking attendance and accessibility dilemmas similar to Daffodil.

### 3.3 Private Accommodations

California students have the right to request private accommodations, for any reason, to change clothes for gym [44]. Accommodations may be separate rooms or simply a curtain drawn across a room. All schools' locker rooms had toilet stalls, but teachers (and laminated signs on each stall in the Daffodil girls' lockers that read “No Dressing in the Restroom”) generally discouraged changing in them. In schools lacking private accommodations (Agapanthus, Daffodil, Marigold), or that only provided accommodations within gendered lockers (Dahlia), TNBA reportedly crossed campus to change in nurse's GNRs—if, as previously noted, they knew about and could access those facilities.

Gym teachers reported that students generally sought privacy. Dahlia and Thistle girls' lockers included privacy stalls for students to change clothes, and a Thistle teacher reported that lines to use these stalls were so long that she had to regularly redirect them to change in the general (non‐private) locker room environment to ensure they would not be late for class. The Thistle teacher noted that a trans girl and a trans boy both use the girl's locker room. She shared that she specifically had to respond to student complaints about the trans boy using the facility. Conversely, Dahlia and Thistle boys' lockers had no such options; there, students reportedly ducked around corners and alcoves for privacy. The Dahlia gym teacher noted that, in deciding to change clothes in alcoves that had doors that open to the outside, these students commonly accepted the risk of exposure in their search for privacy. Similarly, Marigold's girls' lockers provided no stalls; staff claimed that the low walls of the disused gym showers provided partial privacy.

**FIGURE 3:**
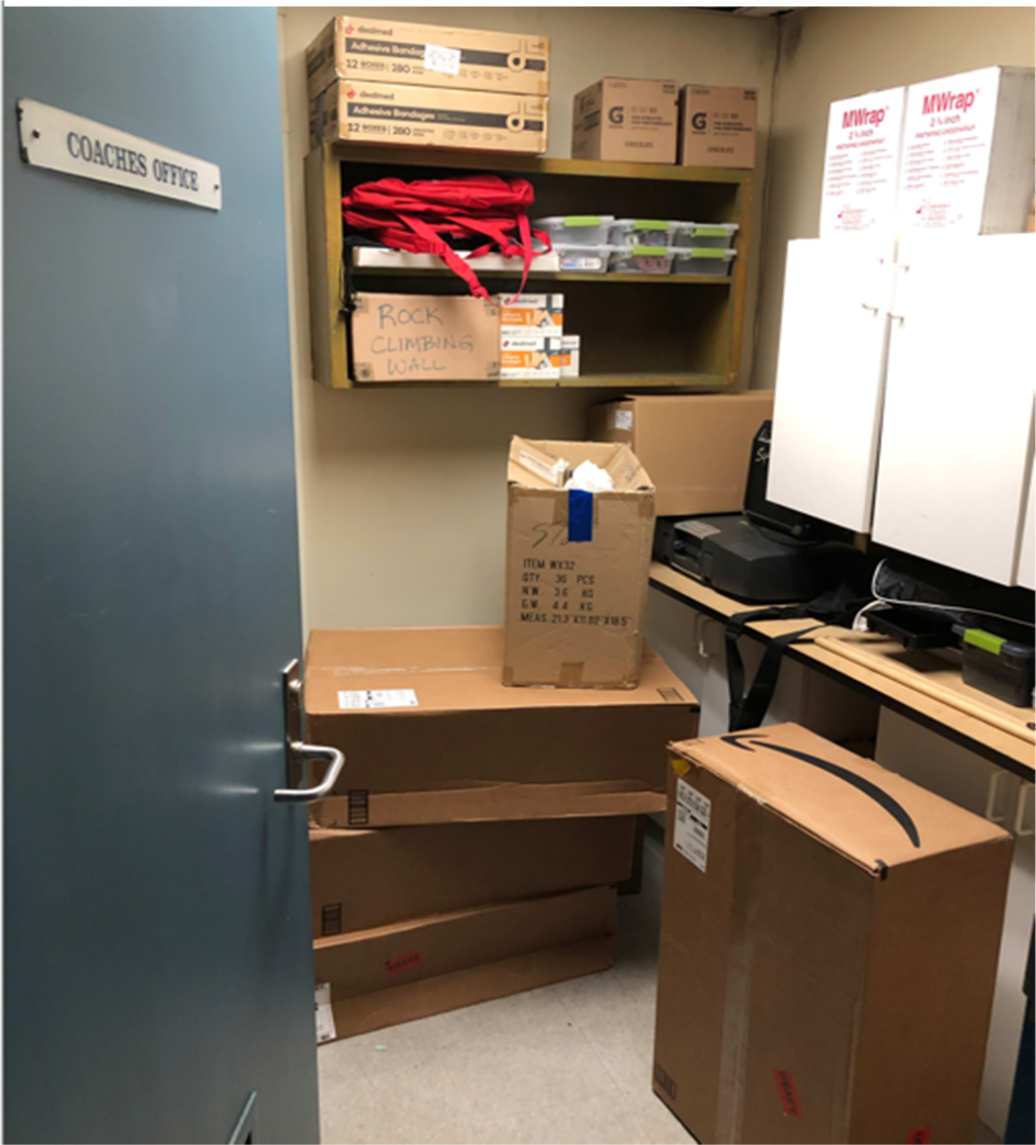
Hibiscus private accommodations.

Dandelion, Hibiscus, and Tiger provided private accommodations near and external to gendered locker rooms and reported that students regularly request and use these facilities. Dandelion's accommodations were in an inner room of the athletic director's office. When the liaison/counselor noted her unfamiliarity with this resource, the athletic director replied that awareness had spread by word of mouth. Hibiscus and Tiger provided coach's offices, both partially used for storage (see Figure 3), and Hibiscus also provided a large, typically empty, Varsity changing room.

Delphinium's lockers were under construction with plans for them to be entirely gender‐neutral, featuring long‐sided lockable changing stalls; that is, a changing stall with walls that extend from floor to ceiling and that have the usual locking mechanism common to any restroom stall. The liaison affirmed the school's commitment to private gender‐neutral construction despite recent community backlash to the announcement of these plans having delayed construction.

### 3.4 Sexual Health Education

The CHYA mandates comprehensive sexual health education delivery affirmative of sexual and gender diversity [23]. Many liaisons lacked details and access to information on CHYA implementation, including syllabi, resulting in limited data for analysis. Tiger and Hibiscus both listed Health as a standalone course required for graduation with an opt‐out option. Tiger's Health syllabus explicitly proclaimed an LGBTQ‐inclusive curriculum.

Marigold, Thistle, Daffodil, and Agapanthus integrated sex education into other courses. Thistle split its 10‐week curriculum between 10th grade World History and Chemistry. Buttercup District required that its schools regularly verify delivery of lessons on gender, sexual orientation, and other topics: while Marigold integrated an LGBTQ‐inclusive 4‐week curriculum into 9th grade biology, Delphinium provided no information to confirm CHYA implementation. The Amaryllis District handbook had a section that detailed CHYA requirements. Daffodil integrated its curriculum into 9th grade biology, but did not specify how many weeks. Finally, despite liaison doubts that its school provides sex education, Agapanthus FGD participants confirmed receipt of “One month or a few weeks toward the end of freshman biology, and we wanted to extend that.”

All Aster District Schools reported adopting district‐approved curriculum in their School Plans for Student Achievement. Unlike Thistle, Dahlia only delivered “booster” events on HIV and healthy relationships to 10th graders; the liaison nonetheless shared an infographic produced by the American Civil Liberties Union of California detailing CHYA requirements. The Dandelion liaison only ever offered that “I think our Sex ed happens in 10th grade history, but I'm not 100% sure,” so Dandelion's CHYA implementation remained unconfirmed.

### 3.5 Innovations

#### 3.5.1 Protecting Intellectual Freedom

Anticipating book ban requests, the Dahlia librarian/liaison proposed a policy for Aster District consideration that outlined complaint and reconsideration processes and emphasized principles of intellectual freedom, with specific reference to guidelines and recommendations of the American Library Association [45]. The proposed policy described library curation procedures and outlined request and adjudication procedures for requests to remove library materials. At the time of the study, the librarian provided a copy of the draft policy, but it had not yet been considered for district adoption. This proactive defense of intellectual freedom at Dahlia High was unique in the sample.

#### 3.5.2 Publishing Information to Facilitate Access

Some schools increased access by providing clear information. The Buttercup District produced a Transgender Student Guide that detailed name change processes and rights to access facilities and sports; guide infographics were displayed throughout Marigold's (but not Delphinium's) campus. Hibiscus published GNR locations in student handbooks.

#### 3.5.3 Health and Wellness Programming

School wellness centers provided targeted supports. Marigold's wellness center regularly hosted a local LGBTQA+ organization to lead events and provide services. Daffodil delivered substantial health programming, including a health center, a clinic providing STI testing, and a wellbeing center featuring LGBTQA+‐specific yoga sessions.

## 4 Discussion

This multiple case study examined the implementation of California TNBA‐protective policies in nine schools. Districts and schools had widely adopted nondiscrimination and Title IX policies inclusive of gender identity—contrary to findings in a similar study with Bay Area schools [16]—though they varied in the posting of these policies on campus. School implementation of name change procedures, gender‐neutral restrooms, private accommodations, and sexual health education varied substantially. This study took place in California—a leader in adopting TNBA protections—and against the backdrop of nationwide movements hostile to TNBA rights [17, 18, 19, 20, 21], underlining the need for evidence on the implementation of these laws.

State, district, and school policy, as well as street‐level enactment, often misalign and indicate a need for oversight, training, and technical assistance. Greater district oversight and school‐district coordination, as in Buttercup District's CHYA reporting mechanisms, may support improved policy implementation. Alternatively, stronger district oversight could have ensured Aster District name change policy implementation. Key implementation issues included data (e.g., how student names are updated, whether names transfer between data systems, and parental access to student information), physical infrastructure (e.g., locations of gender‐neutral restrooms, locks on restroom doors, funding to maintain restrooms), school‐district coordination (e.g., to ensure adoption of sexual health curriculum and permit email changes that align with name changes), pressures to monitor student activity, and student access to information about their rights and services at school.

These barriers reflect commonly cited barriers to the implementation of sexual and gender minority supports in schools, including insufficient resources [46], staff training deficits [47], and infrastructural issues [48]. For example, high schools in this sample sought to control sex, drug, and bullying activity in single‐stall gender‐neutral restrooms by adding locks and requiring staff permission, and thereby made those facilities less accessible to LGBTQ+ students. On the other hand, Delphinium High provided an accessible gender‐neutral restroom that had multiple locking stalls within it, so it could be monitored like any other gendered restroom on campus. Notably, multiple‐stall restrooms are more common than single‐stall restrooms on school campuses, such that the adoption of multiple‐stall gender‐neutral restrooms would be of minimal cost; nominally, the cost of replacing the sign outside the door. Community negative response to the idea of boys and girls sharing restroom spaces can be expected to be the greater threat to such a proposal, as evident in Delphinium High's attempts to expand this concept to their locker room. By focusing on limiting cost, promoting accessibility, and ensuring that these spaces can be more easily monitored than single‐stall restrooms, schools and districts may be able to effectively advocate for multiple‐stall gender‐neutral restrooms on their campuses.

Schools succeeded in implementing policies, often in innovative ways that addressed the implementation issues above. In two key examples, Buttercup District's Transgender Student Guide, with flyers posted on campus at Marigold High, and Hibiscus High's inclusion of the gender‐neutral restroom location in their handbook, facilitated student access to information about their rights and services. Tiger, Hibiscus, and Dandelion exemplified ways to designate private accommodations external to gendered spaces. This study highlights implementation shortcomings and successes, indicating that TNBA‐protective policy implementation is feasible with greater coordination, training, and funding between the State Department of Education, districts, and schools.

State laws like the CHYA and SSOA can be amended to support implementation, especially considering that most of the laws reviewed in this study lack any appropriations, implementation timelines, or staff training requirements. Variation in health curriculum delivery reflects vague legal requirements in the CHYA. While the CHYA mandates coverage of specific content, including sexual and gender diversity, it does not mandate content delivery in a standalone course versus integration into other courses or events, nor does it set the number of hours of instruction [23]. Comprehensive sexual health education is associated with increased odds of delayed sex and reduced odds of teen pregnancy [49, 50, 51]. Despite this, and for lack of stronger CHYA requirements on the number of hours of instruction, some schools in this study provided little to no sexual health instruction. Implementation of California privacy and nondiscrimination protections, like the SSOA, can be supported by stronger requirements that private accommodations be designated in gender‐neutral spaces. Such policy changes to extend TNBA protections have been accomplished recently: SB 790 requires equitable provision of unlocked and unobstructed GNRs and that GNRs be included in modernization projects [27]. Short of attaining modernization funds, many schools—like those detailed in this study, such as Dahlia—can be expected to struggle to meet the costly requirements of SB 790 given the infrastructural, financial, and training barriers identified in this study.

### 4.1 Implications for School Health Policy, Practice, and Equity

Study findings emphasized successes, innovations, and challenges faced by policymakers and educators in implementing TNBA‐protective policies. Cases exemplify the feasibility of implementation of these policies with the establishment of clear and accessible procedures, training and technical assistance, and funding and infrastructural support. Findings inform the adoption of name change policies, provision of GNRs and private accommodations, and delivery of sexual health education. Key recommendations include gender transition planning and publishing transgender student policies, including the right to update records and the locations of GNRs and private accommodations, in handbooks, flyers, and on websites. While the ERAA requires that all single‐stall restrooms be gender‐neutral, California schools preparing for the 2026 deadline to implement SB 760 [27] will need to offer more GNRs with clear signage in less supervised, more accessible, and central locations. Actual infrastructure updates are extremely expensive; though, GNR updates eligibility under California SB 670 for modernization project proposals may be one potential resource to address this barrier in California [27].

### 4.2 Limitations

As an explorative qualitative case study, findings detailed non‐generalizable, temporally, and contextually specific snapshots of policy implementation in nine schools. School differences, information access, and liaison roles may have biased findings. Schools differed in student body size, the proportion of students of color, and the proportion of students eligible for reduced‐price lunch (see Table 1). The small number of participating schools precludes any statistical inference regarding student body demographics and policy findings. The two schools that most consistently implemented trans‐protective policies and protections had notably different student bodies: where the Hibiscus High student body was below average for this study (1700+ students), mostly students of color (90%–100%), and eligible for reduced‐price lunch (90%–100%) and Tiger High was larger (2600+ students) and had the smallest proportions of students of color (60%–70%) and students eligible for reduced‐price lunch (20%–30%) in the sample. For liaison roles, the Dahlia liaison/librarian told researchers about her own efforts to have the Aster District adopt an intellectual freedom policy. On the other hand, the Dandelion liaison did not know and never confirmed the school's health curriculum delivery, limiting conclusions that could be made about that school. Schools within Aster, Amaryllis, and Buttercup Districts could be compared, whereas single school participation from the Lily and Mallow Districts prevented within‐district comparisons. The observation protocol was not previously piloted; protocol co‐development with liaisons may have improved relevance to each school. FGDs' testimony contextualized findings in only three schools; student testimony from other schools would have aided triangulation and enriched findings. While the RCT interventions were not expected to affect school policies, given the timing of the interventions, participation in the RCT study may have biased staff responses in this case study.

Despite these limitations, the explorative qualitative case study approaches of incorporating and triangulating many data sources, following leads, and seeking feedback fostered deep and rich insights into each school and strengthened the trustworthiness of study findings [52]. Besides this study and another in Bay Area districts [16], no study has surveyed TNBA‐protective policy adoption and implementation statewide or in rural areas. Study findings may resonate with educator experiences in California and elsewhere in the U.S., who may ask how their own schools are implementing these protections.

## 5 Conclusion

TNBAs experience violations of privacy, disaffirmation of gender identities, reduced facility access, and curricular exclusion in school. These experiences contribute to increased risks of violence [2, 6], school dropout [11], and suicide ideation [2, 12]. Study findings illustrated variable implementation of California policies to protect TNBA rights to name changes, restrooms, private accommodations, and comprehensive sexual health education. Despite this variation, notable successes highlighted the feasibility of TNBA‐protective policy implementation. Findings that infrastructure, financial, training, and technical barriers inhibit school policy implementation likely resonate with educators and TNBA experiences state‐ and nationwide. Future research may investigate these policies statewide, in rural areas, and in other states with and without these protections, and should incorporate staff and student voices. For example, multi‐state comparisons have extended understanding of how state policies shape TNBA health [53]. Such investigations with adolescents are needed to understand the implications of policy on TNBA health. Key recommendations include promoting access by publishing TNBA resources, setting stricter state standards for sexual health education, and extending statewide training and technical assistance to support policy implementation.

## Funding

This work was supported by the National Institute on Minority Health and Health Disparities (R01MD016082‐04) and intramural funding from the University of Southern California Office of the Provost, including a dissertation fellowship and two research grants.

## Ethics Statement

This study was approved by the University of Southern California Internal Review Board on February 3, 2023, with Study ID UP‐22‐00773.

## Conflicts of Interest

The authors declare no conflicts of interest.

## Supporting information


**Table S1:** Documents received and information observed from each school.

## Data Availability

The data that support the findings of this study are available on request from the corresponding author. The data are not publicly available due to privacy or ethical restrictions.
