# Supplementary material for: Adoption and Implementation of California State Transgender and Nonbinary Protections in Los Angeles Area High Schools
Source: J Sch Health. 2025 Dec 10;96(1):e70100. doi: 10.1111/josh.70100 (PMC12696403; doi:10.1111/josh.70100)
Supplement: Supplementary file 1 — Table S1: Documents received and information observed from each school. [file JOSH-96-0-s001.docx]

| **Supplemental Table 1, Documents Received and Information Observed from Each School** | | | | | | | | |
| --- | --- | --- | --- | --- | --- | --- | --- | --- |
| District | School | Handbook | Registrar^a^ | School Map | Course List | Syllabi^b^ | SPSA^c^ | SARC^d^ |
| Aster | Dandelion | ✓ | ✓ | ✓ | ✓ |  | ✓ | ✓ |
|  | Thistle | ✓ | ✓ | ✓ | ✓ | ✓ | ✓ | ✓ |
|  | Dahlia | ✓ | ✓ | ✓ | ✓ | ✓ | ✓ | ✓ |
| Amaryllis | Agapanthus | ✓ | ✓ | ✓ | ✓ |  | ✓ | ✓ |
|  | Daffodil | ✓ | ✓ | ✓ | ✓ |  |  | ✓ |
| Buttercup | Delphinium | ✓ | ✓ | ✓ |  | ✓ |  |  |
|  | Marigold | ✓ | ✓ | ✓ | ✓ | ✓ |  | ✓ |
| Mallow | Hibiscus | ✓ | ✓ | ✓ | ✓ | ✓ |  | ✓ |
| Lily | Tiger | ✓ | ✓ | ✓ | ✓ | ✓ | ✓ | ✓ |
| ^a^ Registrar includes any training document, email communication, or observation of registrar procedures  ^b^ Syllabi includes sexual health education syllabi and/or curriculum descriptions  ^c^ SPSA: School Plan for Student Achievement, a report created by California schools that details school curriculum, facilities, and progress toward goals  ^d^ SARC: School Accountability Report Card, a detailed report created by California schools on key educational outcomes (e.g., reading levels, attendance) and disparities | | | | | | | | |
